# Spatial distribution and geographical heterogeneity factors associated with households' enrollment level in community-based health insurance

**DOI:** 10.3389/fpubh.2024.1305458

**Published:** 2024-05-17

**Authors:** Addisalem Workie Demsash

**Affiliations:** Department of Health Informatics, Debre Berhan University, Asrat Woldeyes Health Science Campus, Debre Birhan, Ethiopia

**Keywords:** health insurance, households, enrollment, spatial analysis, geographical analysis

## Abstract

**Background:**

Healthcare service utilization is unequal among different subpopulations in low-income countries. For healthcare access and utilization of healthcare services with partial or full support, households are recommended to be enrolled in a community-based health insurance system (CBHIS). However, many households in low-income countries incur catastrophic health expenditure. This study aimed to assess the spatial distribution and factors associated with households' enrollment level in CBHIS in Ethiopia.

**Methods:**

A cross-sectional study design with two-stage sampling techniques was used. The 2019 Ethiopian Mini Demographic and Health Survey (EMDHS) data were used. STATA 15 software and Microsoft Office Excel were used for data management. ArcMap 10.7 and SaTScan 9.5 software were used for geographically weighted regression analysis and mapping the results. A multilevel fixed-effect regression was used to assess the association of variables. A variable with a *p* < 0.05 was considered significant with a 95% confidence interval.

**Results:**

Nearly three out of 10 (28.6%) households were enrolled in a CBHIS. The spatial distribution of households' enrollment in the health insurance system was not random, and households in the Amhara and Tigray regions had good enrollment in community-based health insurance. A total of 126 significant clusters were detected, and households in the primary clusters were more likely to be enrolled in CBHIS. Primary education (AOR: 1.21, 95% CI: 1.05, 1.31), age of the head of the household >35 years (AOR: 2.47, 95% CI: 2.04, 3.02), poor wealth status (AOR: 0.31, 95% CI: 0.21, 1.31), media exposure (AOR: 1.35, 95% CI: 1.02, 2.27), and residing in Afar (AOR: 0.01, 95% CI: 0.003, 0.03), Gambela (AOR: 0.03, 95% CI: 0.01, 0.08), Harari (AOR: 0.06, 95% CI: 0.02, 0.18), and Dire Dawa (AOR: 0.02, 95% CI: 0.01, 0.06) regions were significant factors for households' enrollment in CBHIS. The secondary education status of household heads, poor wealth status, and media exposure had stationary significant positive and negative effects on the enrollment of households in CBHIS across the geographical areas of the country.

**Conclusion:**

The majority of households did not enroll in the CBHIS. Effective CBHIS frameworks and packages are required to improve the households' enrollment level. Financial support and subsidizing the premiums are also critical to enhancing households' enrollment in CBHIS.

## Background

Many countries face significant challenges in financing healthcare (1, 2). Health services are unavailable and unaffordable for households of poor wealth status (3, 4), leading to unequal access to healthcare across different subpopulations (5). The demands of medical care expenditure and utilization are different between inpatients and outpatients (6). Poor quality, financial constraints, absence of pre-payment financial arrangements (7, 8), and direct user-fee charges (9) were major factors for the low utilization of healthcare services. Globally, 50% of the world's population cannot access needed health services, while 100 million people are pushed into extreme poverty each year because of health expenses (10). Every year, 150 million people suffer from financial catastrophes (11). Thus, the access to and utilization of healthcare services among households is low (12, 13).

In low- and middle-income countries, over 40% of total health expenditure is done through out-of-pocket payments (OPP), which has resulted in a scarcity of funds to be spent on health (14). It has been reported that 5.5%, 15%, and 2.6% of households in Mongolia (15), Burkina Faso (16), and Uganda (17) suffer from catastrophic health expenditure, respectively. Up to 90% of healthcare expenditure catastrophes occurred in low-income countries (18, 19), and OPP accounts for 37% of the total spending on healthcare (20).

Social health insurance (SHI) is a scheme that offers an opportunity for healthcare finance by raising and pooling funds (21) to provide affordable, cost-effective, and equitable healthcare services (22–24). Community-based health insurance schemes (CBHIS) are one of the mechanisms for raising funds for access to healthcare services (25). A CBHIS is a type of insurance in which households or household members register and pay a premium to the health insurance system. The head of the households or one of the members is registered in the proposed CBHIS regardless of the amount paid for partial or full health insurance coverage (41). Thus, the medical and surgical expenses would be covered by the health insurance system, which allows the members of the household to access health services without any financial hardship (10).

CBHIS has been advocated by the World Health Organization (WHO) to achieve universal healthcare coverage and ensure access to healthcare services (26). CBHIS is a primary agenda for health reform in many countries for universal health coverage (27, 28), and it guarantees individuals' access to healthcare services (29, 30). Moreover, CBHIS is designed for the agricultural and other informal sectors to enhance productivity and provide food security (31). The enrollment process in health insurance schemes is voluntary, and it is a complementary or alternative source of healthcare finance (32). Sometimes, people may be forced to pay CBHIS premium based on the principle of social solidarity (23).

Despite health insurance being focused on whole populations, it is used more by older adult people due to low incomes and the absence of social security (6). Hence, CBHIS has not reached the level of universal coverage (5). Moreover, health service utilization is different among people in different health insurance schemes (33) and CBHIS is ineffective in many countries (34). For instance, 45% of people are not covered by CBHIIS in China (35).

In Ethiopia, according to the 2016 Ethiopian Demographic and Health Survey (EDHS), health insurance coverage is shallow (10); more than 94% of households are not covered by health insurance (36). Primary studies in Ethiopia show that only 12.8% of the households are enrolled in CBHIS in Sidama (37) and 33.30% of the households in south Omo (38), while 77.9% of the population complied with CBHIS requirements in southeast Ethiopia (39). Educational status of the household, monthly income, sex (25), media exposure, age, occupation, wealth status, size of family members (10), knowledge and attitudes toward CBHIS, and trust in CBHIS management (37) are factors for the enrollment of households in CBHIS.

A literature search shows that studies on the assessment of household enrollment in CBHIS are scarce. The enrollment of households in the cold areas (low) in CBHIS has not been identified in Ethiopia. If geographical weighted regression analysis is employed for insufficient empirical evidence, policymakers and stakeholders cannot decide who gets benefits from the system and who is left behind. Hence, the policymakers require potential evidence as a source for future enhancement of CBHIS coverage and to take proper action. Identifying geographical variations of households' enrollment in CBHIS is very important to prioritize and design a framework and install packages in the CBHIS programs in the specific target location. Therefore, this study aimed to explore the spatial distribution and factors associated with households' enrollment level in CBHIS.

## Methods

### Study design and setting

A community-based cross-sectional study design was conducted across the nine regions of Ethiopia. Ethiopia is located in the Horn of Africa and bordered by Eritrea to the north, Djibouti and Somalia to the east, Sudan and South Sudan to the west, and Kenya to the south. Ethiopia comprises nine regional states with two administrative cities (10).

### Data source

The 2019 Ethiopian Mini Demographic and Health Survey (EMDHS) dataset was used from the Measure Demographic and Health Survey (DHS) program website (https://dhsprogram.com). The survey was conducted by the Ethiopian Public Health Institute (EPHI) in collaboration with the Central Statistical Agency (CSA). According to the EMDHS report, the survey was conducted from 21 March to 28 June 2019. Shapefiles were downloaded from https://africaopendata.org.

### Sampling techniques and study population

A two-stage stratified cluster sampling was used. Each region was stratified into urban and rural areas. In the selected enumeration areas, a household listing operation was done, and the results were used as a sampling frame for household selection in the second stage. Finally, a fixed number of households per cluster were selected. Samples from enumeration areas were selected independently in each stratum through implicit stratification and equal proportional allocation. The details about the methodology are found from the 2019 EMDHS reports (40).

### Variables of the study

#### Individual and community-level independent variables

Sociodemographic characteristics of households, such as wealth status, and household-related variables such as age, sex, educational status, and household media exposures were considered individual-level variables, whereas the place of residence and region were used as community-level variables.

### Dependent variable

Households' enrollment level in the CBHIS is dependent variable.

### Operational definition

*Households' enrollment level in CBHIS :* The households were considered as enrolled in the CBHIS if they had registered for partial or full health service cost waiver under the proposed CBHIS, and these households were coded as “1”. Otherwise, households that had not enrolled in CBHIS were coded as “0” (41).

*Media exposure:* If the households had either radio or television or both, then we considered that they are exposed to media, and if they did not have either of them, then we considered that they are unexposed to media (42).

### Data management and processing

The STATA version 15 software and Microsoft Office Excel were used for data management. ArcMap version 10.7 software was used for spatial autocorrelation detection and interpolations of households' enrollment levels in CBHIS in Ethiopia.

*Global spatial autocorrelation:* The global spatial autocorrelation (Global Moran's I) statistic measure was used to assess whether the households' enrollment level in CBHIS was dispersed, clustered, or randomly distributed in Ethiopia (43). Moran's I values close to −1, close to +1, and zero (0), respectively, indicate a dispersed, clustered pattern and random distribution (44, 45) of households' enrollment level in CBHIS. The Z-scores and *p-*values were used to determine the hot and cold areas.

*Spatial interpolation:* Unsampled areas were predicted by the spatial interpolation of the households' enrollment level in CBHIS based on sampled EAs. For the prediction of unsampled EAs, we used the radial basis function interpolation technique.

*Spatial scan statistics:* SaTScan version 9.5 software was used for local cluster detection (46). We employed purely spatial Bernoulli-based model scan statistics to determine the geographical locations of significant clusters with high rates of household enrollment level in CBHIS (47). Those households that were enrolled in CBHIS were treated as cases, and those that had not enrolled were taken as controls to fit the Bernoulli model for the scanning window that moves across the study area. The default size of < 50% of the population was used as an upper limit, allowing both small and large clusters to be detected and ignoring clusters that contained more than the maximum limit because of the circular shape of the window. The log-likelihood ratio (LLR) was used to determine whether the number of observed cases within the potential cluster was significantly higher than expected. The circle with the maximum LLR was defined as the most likely cluster, and it was then compared with the overall distribution of maximum values. The significant clusters were assigned *p*-values and ranked based on their LLR value based on the 9999 Monte Carlo replications (48).

### Geographically weighted regression

Both the ordinary least squares (OLS) model and the geographical weighted regression (GWR) model were considered for model fitness comparison. The extracted predictor variables were fitted into the two models. Adjusted R^2^ and Akaike's information criterion (AIC) were used to compare and determine the best-fit model for local parameter estimation. Variance inflation factors (VIF) were used for multicollinearity checking by the same dependent and explanatory variables in the OLS model. Variables with VIF values of > 0.7S were considered redundant.

A GWR analysis model was used to determine the aggregate effects of each explanatory variable for households' enrollment in the CBHIS. A GWR model was used for the estimation of the local parameter to reflect changes that occur over space in the spatial association between a dependent variable and explanatory variables, as well as for relaxing the geographical independence of explanatory variables (44, 49). Therefore, the following GWR model linear assumption of mathematical equations (model structure) was written. *Wi* = β (*Xi, Yi*)+*ZHα*(*Xi, Yi*)*Zi*+*i*, where Wi is the response variable, (Xi, Yi) are the geographical coordinates of point i, β is the intercept at the (Xi, Yi) coordinate, α e coefficient of the covariate Z at the (Xi, Yi) coordinate, and ϵi is the random error term. Finally, the explanatory variables with a *p* < 0.05 were considered as stationary significant factors for households' enrollment level in CBHIS.

### Multilevel fixed-effect logistic regression analysis

Since the nature of the EMDHS dataset was hierarchical, the records within the cluster might be correlated, which disturbs the assumption of independence. A biased statistical report might be generated by fitting a model with correlated data. Therefore, multilevel mixed-effect logistic regression analysis was assumed to be have been used to generate and report good results. To assess the correlation between the clusters, four models have been set: model A (a null model that assesses the households' enrollment level in CBHIS within the cluster); model B (contains individual-level variables); model C (contains community-level variables); and model D (the aggregate model of models B and C). The intraclass correlation coefficient (ICC) was calculated to check the correlation within the cluster. If the ICC value is >0.25, the data are fitted for a multilevel fixed-effect logistic regression model (50, 51). An LLR was used for model comparison, and the model with the highest value was taken as the best-fit model to solve the correlation within the cluster (52). In the multilevel fixed-effect logistic regression analysis, a *p* < 0.05 and 95% CIs were used to assess the strength of the association between independent and outcome variables.

## Results

### Sociodemographic characteristics of the study

A total of 40,659 weighted sampled households were used in this study. Four out of 10 (40.5%) and one-fifth (20.1%) of the households were from the Oromia region and South Nation Nationality and People of the Region (SNNPR), respectively. The majority (72.9%) of the households were from rural areas of the country. Of the sample, 46.9% of household heads were not educated, and only four out of 10 (42.1%) of the household heads had completed primary education. Four out of 10 (40.3%) households were in the rich wealth index status. The majority (76.2%) of household heads were more than 34 years of age, and 82.3% of household heads were men. More than seven out of 10 (70%) of households had no television (TV) or radio (Table 1).

**Table 1:** Sociodemographic characteristics of households.

**Variable**	**Category**	**Frequency (n)**	**Percent (%)**
Place of residence	Urban	11,019	27.1
	Rural	29,640	72.9
Region	Tigray	2,521	6.2
	Afar	407	1.0
	Amhara	8,294	20.4
	Oromia	16,467	40.5
	Somali	2,480	6.1
	Benishangul Gumuz	447	1.1
	SNNPR	8,172	20.1
	Gambela	163	0.4
	Harari	122	0.3
	Addis Ababa	1,342	3.3
	Dire Dawa	244	0.6
Educational status of household heads	No education	19,069	46.9
	Primary	17,117	42.1
	Secondary	3,090	7.6
	Higher	1,382	3.4
Age of household head	15–24	1,651	4.06
	25–34	8,010	19.7
	>=35	30,982	76.2
Sex of household head	Male	33,462	82.3
	Femen	7,197	17.7
Households' wealth status	Poor	16,142	39.7
	Middle	8,132	20.0
	Rich	16,386	40.3
Household has radio	No	29,274	72.0
	Yes	11,385	28.0
Households have Tv	No	34,235	84.2
	Yes	6,424	15.8

### Spatial distribution of health insurance coverage in Ethiopia

**Figure 1:**
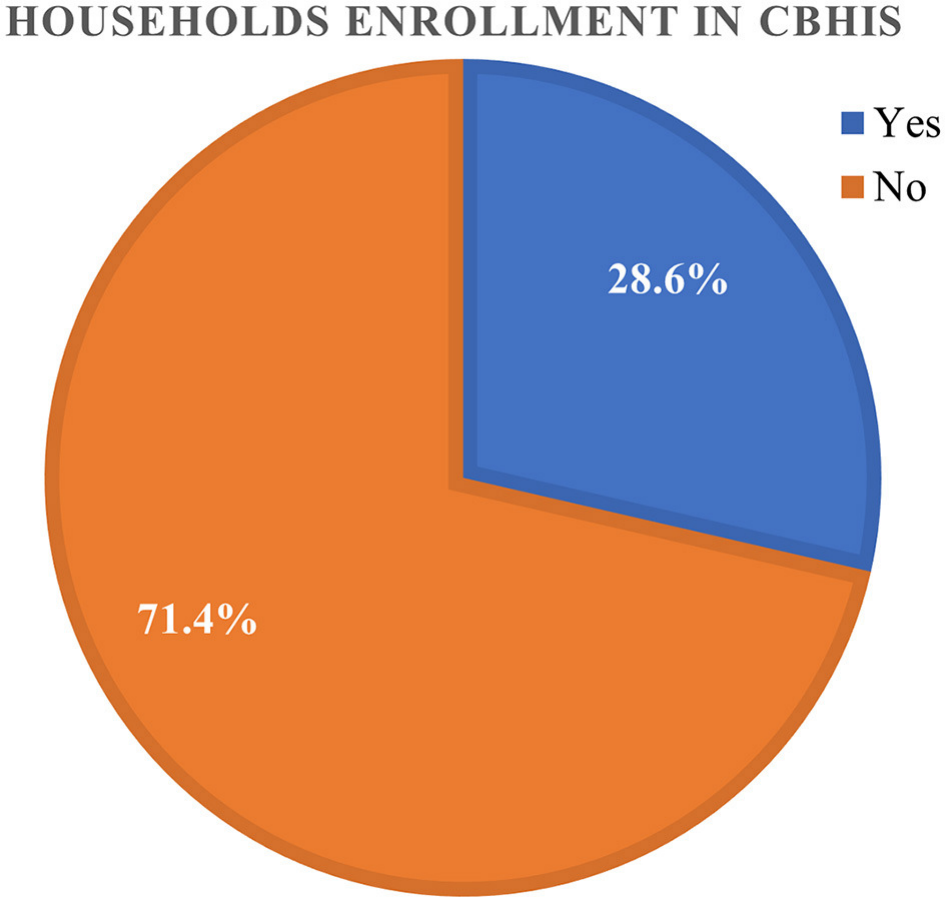
Households' enrollment level in CBHIS.

**Figure 2:**
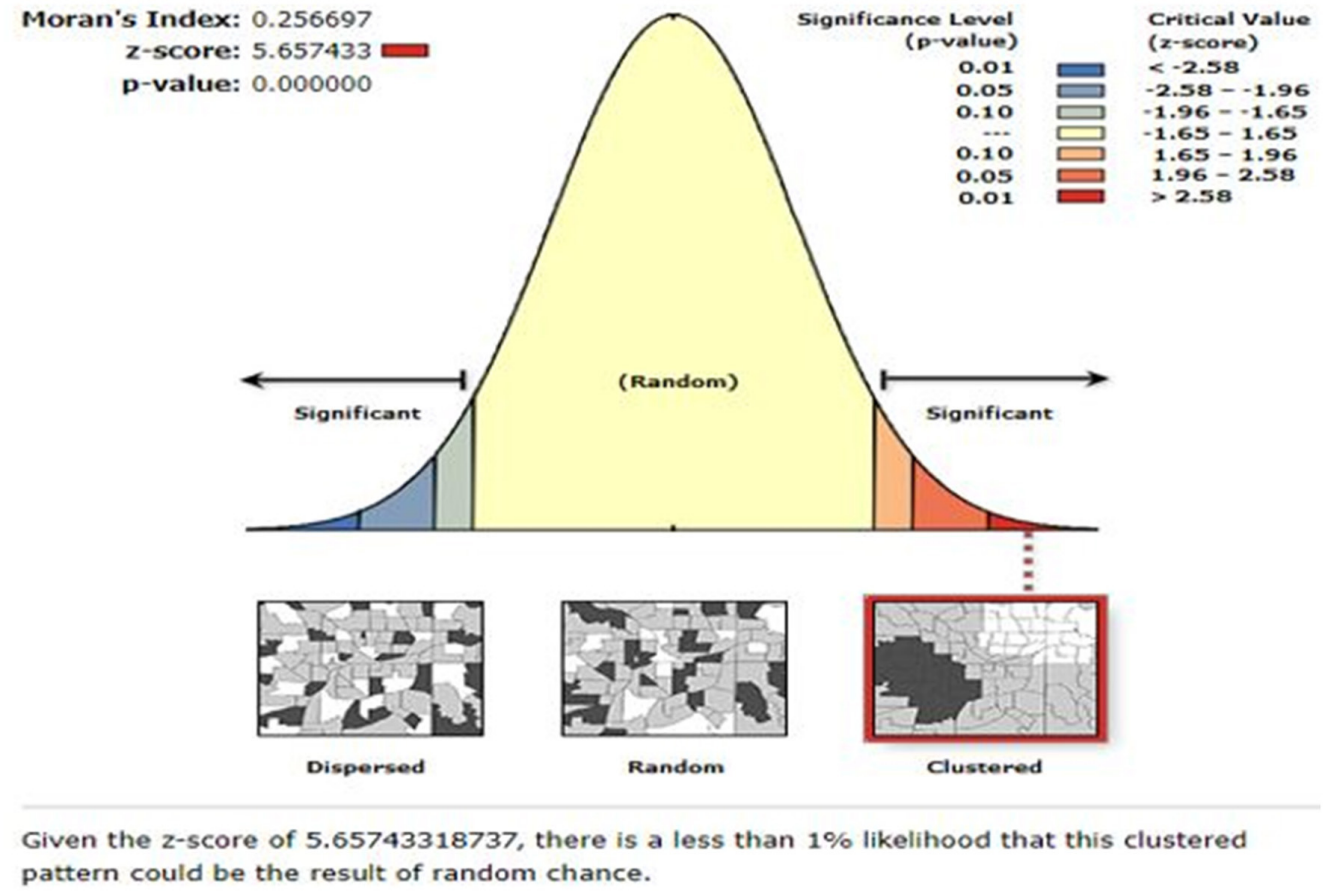
Spatial autocorrelation report of households' enrollment level in CBHIS.

As stated in Figure 1, 28.6% (95% CI: 28.16–29.04%) of the households in Ethiopia had good levels of enrollment in CBHIS. The spatial distribution of households' enrollment level in CBHIS was not random in Ethiopia (Global Moran's I = 0.256697, *p* < 0.00001). The spatial autocorrelation report indicated that the spatial distribution of households' enrollment in CBHIS was significantly clustered across the regions of Ethiopia (Figure 2).

**Figure 3:**
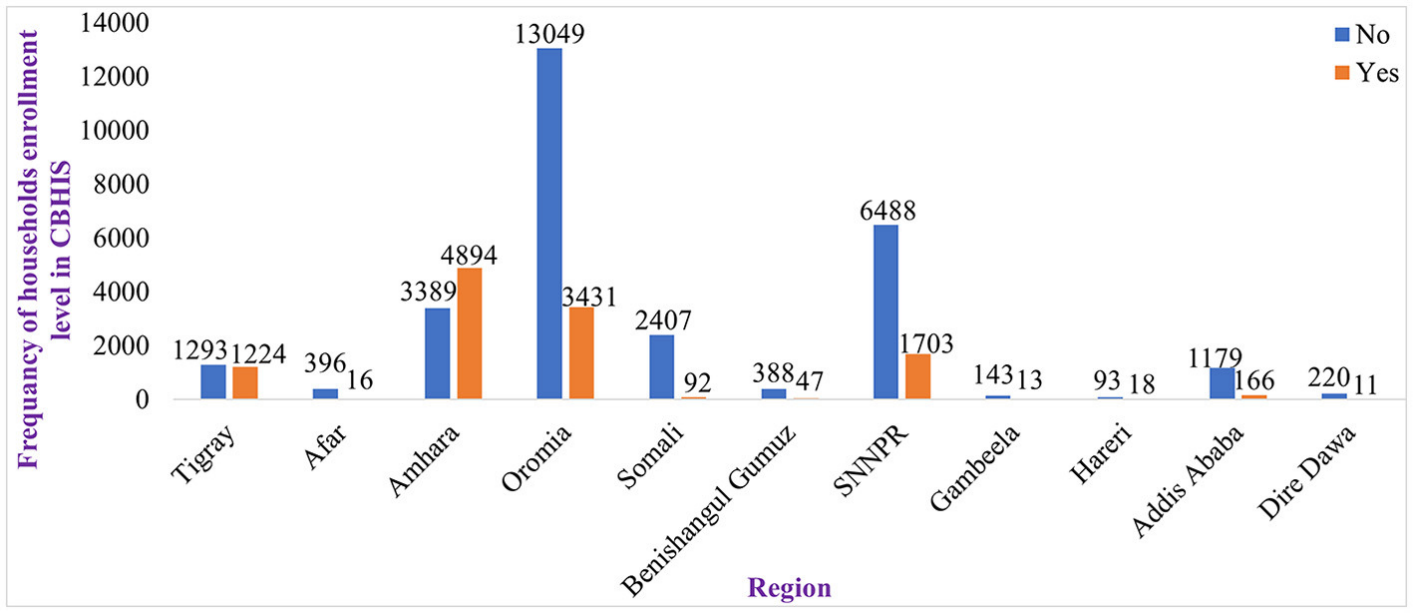
Households' enrollment level in CBHIS stratified by region.

**Figure 4:**
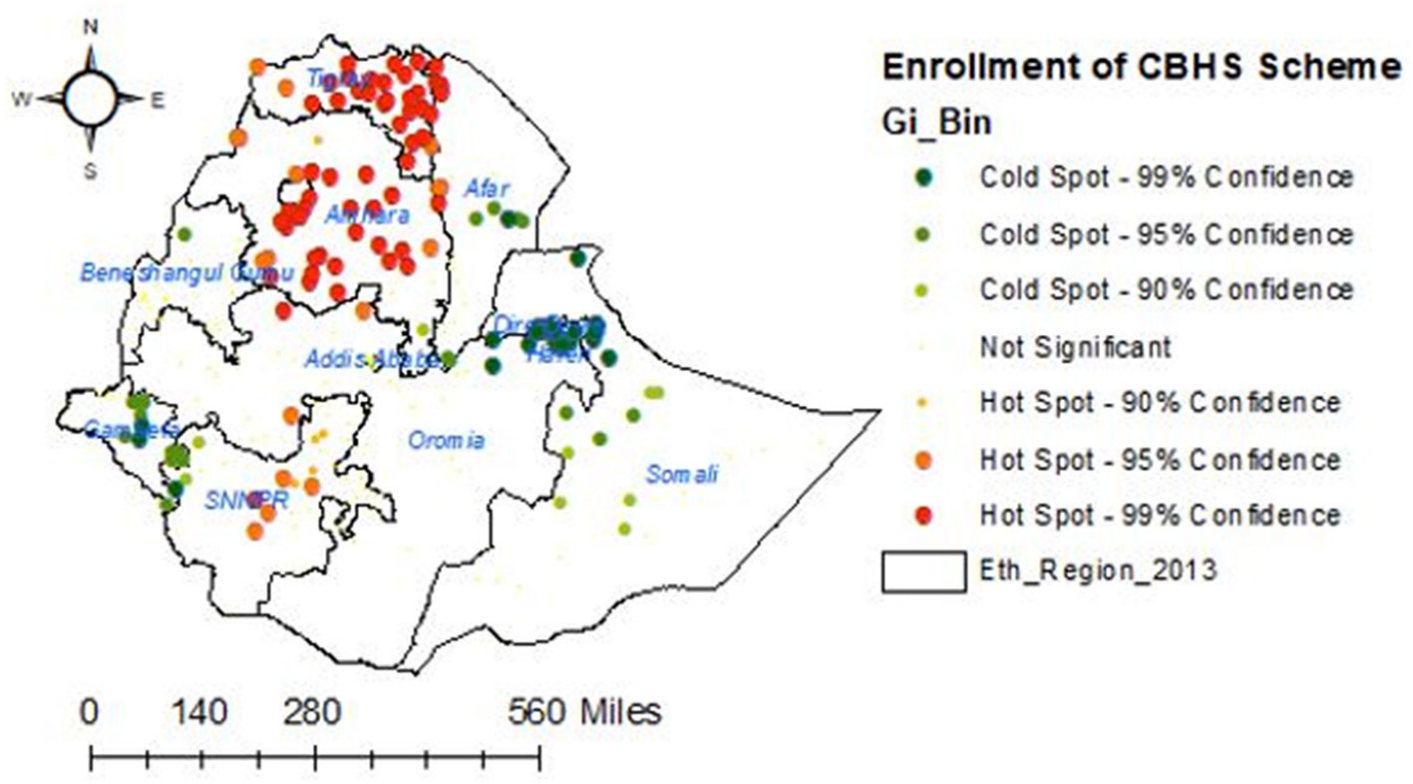
Hot spot analysis for households' enrollment levels in CBHIS in Ethiopia.

A good level of households' enrollment in CBHIS was observed in the regional states of Ethiopia such as Amhara, Tigray, SNNPR, and Oromia, whereas households' enrollment levels in CBHIS in the Benishangul Gumuz, Harari, Afar, and Dire Dawa regions were low (Figures 3, 4).

### Interpolation of households' enrollment level in CBHIS

**Figure 5:**
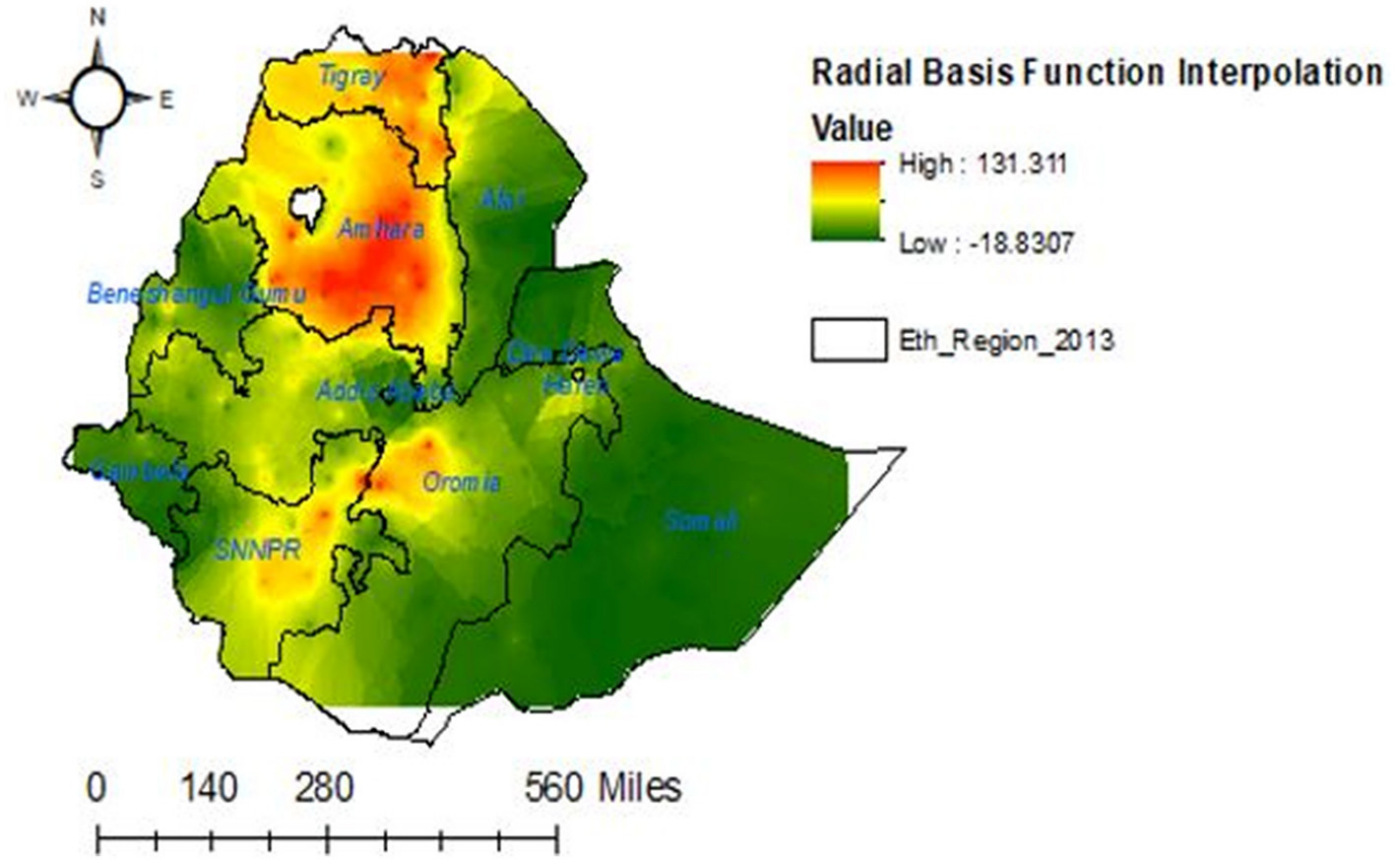
Interpolation of households' enrollment levels in CBHIS in Ethiopia.

The radial basis function interpolation method was employed to predict the households' enrollment level in CBHIS. The interpolation result indicated that the risk of households' enrollment in CBHIS was less likely to occur in the Amhara, Tigray, SNNPR, and Oromia regions, whereas high risk of households' enrollment in CBHIS was more likely observed in Benishangul Gumuz, Gambela, Somalia, Afar, Harari, and Dire Dawa regions (Figure 5).

### Spatial SaTScan analysis

**Figure 6:**
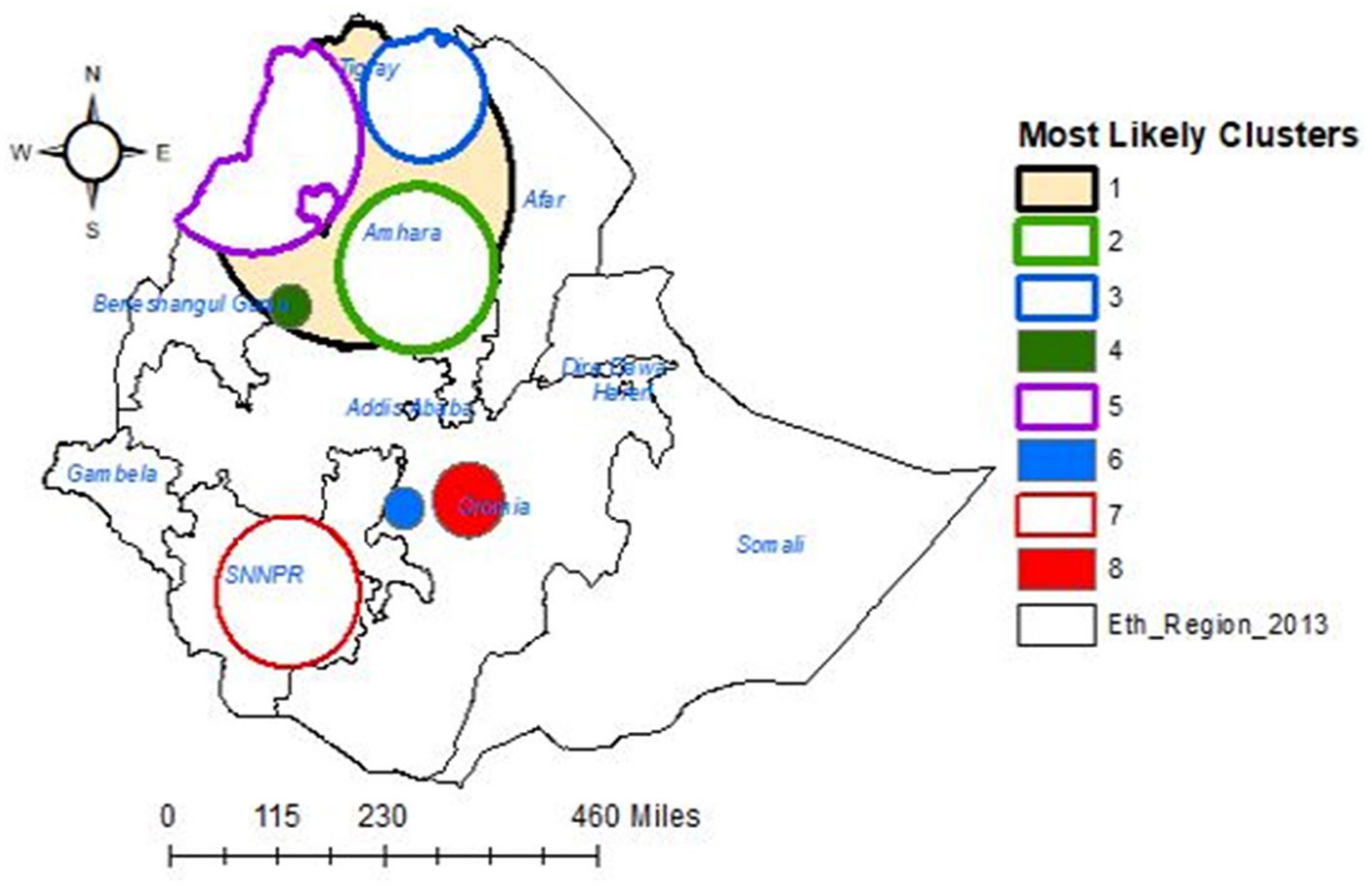
SaTScan analysis of households' enrollment levels in CBHIS in Ethiopia.

A total of 126 significant clusters for households' enrollment levels in CBHIS were detected. Of these, 65, 15, and 19 clusters were primary, secondary, and tertiary clusters, respectively. The primary clusters were located at 12.322718 N, 37.959425 E within a 265.25 km radius in the north and northwest parts of Ethiopia. The secondary clusters were located at 11.072967 N, 38.884163 E within a 135.21 km radius in the southern part of the Amhara regional state of the country. Households in the primary, secondary, and tertiary clusters were 3.58, 4.24, and 2.80 times more likely to have good levels of enrollment in CBHIS than households outside the clusters (Table 2, Figure 6).

**Table 2:** Significant spatial scan statistics clusters for households' enrollment levels in CBHIS, 2019 EMDHS dataset.

**Types of clusters**	**Detected cluster**	**Coordinate/ Radius**	**Population**	**Case**	**RR**	**LLR**	**P-value**
Primary	83, 82, 57, 84, 56, 78, 58, 59, 54, 81, 74, 61, 60, 62, 75, 53, 9, 22, 18, 70, 76, 23, 65, 20, 71, 7, 13, 2, 8, 14, 21, 72, 55, 24, 85, 1, 5, 51, 79, 63, 12, 19, 52, 11, 165, 80, 46, 29, 17, 44, 6, 25, 73, 162, 77, 66, 36, 3, 64, 45, 16, 10, 163, 4, 67	(12.32271 N, 37.959425)/ 265.25 km	8094	1700	3.58	2156.4	< 0.001
Secondary	65, 51, 63, 60, 66, 61, 58, 67, 71, 64, 62, 70, 73, 76, 68	(11.07296 N, 38.884163)/ 135.21 km	1773	1381	4.24	1393.2	< 0.001
Tertiary	2, 14, 11, 13, 17, 12, 23, 5, 7, 16, 3, 25, 10, 1, 36, 20, 9, 24, 15	(13.64134 N, 38.981085)/ 106.17 km	2403	1276	2.80	644.5	< 0.001
Fourth	77, 80, 79	(10.5112 5N, 36.855595)/ 36.15 km	336	278	4.04	296.2	< 0.001
Fifth	85, 55, 21, 84, 4, 56, 22, 82, 74, 75	(12.98563 N, 36.239465)/ 188.33 km	1284	655	2.55	292.3	< 0.001
Sixth	116, 203	(7.531183 N, 38.662596)/ 34.42 km	352	260	3.60	227.8	< 0.001
Seventh	173, 196, 192, 198, 199, 204, 191, 197, 190, 189	(6.272978 N, 36.862733)/ 124.08 km	1481	654	2.20	208.4	< 0.001
Eight	103, 104	(7.648661 N, 39.688764)/ 61.78 km	345	214	3.00	137.3	< 0.001

### Individual and community-level factors for household's enrollment level in CBHIS

As stated above, ICC and LLR were used for multilevel fixed-effect analysis. A comparison revealed that 68.7% of the ICC values in Model D confirmed that there was a significant correlation among respondents within the cluster on households' enrollment level in CBHIS. Additionally, model D was the best-fit model as its LLR score was higher (−12941.701) than other models shown in Table 3. Therefore, a multilevel fixed-effect logistic regression analysis model was employed to determine the correlations within the cluster among respondents based on their enrollment in CBHIS. In the multilevel fixed-effect logistic regression analysis, educational status, age of the household head, wealth status, media exposure, and regions were statistically associated with households' enrollment in CBHIS.

**Table 3:** Multilevel fixed-effect logistic regression analysis of factors associated with households' enrollment in CBHIS in Ethiopia.

**Variables**		**Model A**	**Model B**	**Model C**	**Model D**
			AOR (95% CI)	AOR (95% CI)	AOR (95% CI)
Education status	Primary		1.23 (0.05, 1.31)		1.21 (0.04, 1.31)
	Secondary		1.26 (1.89, 2.46)^a^		1.25 (1.89, 2.44)^b^
	Higher		0.89 (0.35, 1.85)		0.88 (0.34, 1.84)
	No formal education		1		1
Age of the household head	25–34 years		1.90 (0.54, 2.33)	-	1.90 (0.53, 2.32)
	≥35 years		2.48 (1.04, 3.03)^a^	-	2.47 (1.04, 3.02)^b^
	15–24 years		1	-	1
Wealth status	Poor		0.32 (0.12, 1.82)^a^	-	0.31 (0.12, 0.81)^b^
	Middle		0.13 (0.18, 1.97)	-	0.42 (0.17, 1.85)
	Rich		1		1
Household head sex	Female		0.91 (0.83, 0.99)^a^	-	0.92 (0.84, 1.002)
	Male		1		1
Media exposure	Yes		1.36 (1.02, 2.28)^a^	-	1.35 (1.02, 2.27)^a^
	No		1		1
Region	Afar		-	0.01 (0.003, 0.02)^a^	0.01 (0.003, 0.03)^b^
	Amhara		-	1.5 (0.60, 3.89)	1.55 (0.60, 3.97)
	Oromia		-	0.14 (0.06, 1.37)	0.14 (0.05, 1.36)
	Somali		-	0.6 (0.02, 1.12)	0.5 (0.02, 1.11)
	Benishangul		-	0.3 (0.1, 1.09)	0.3 (0.1, 1.08)
	SNNPR		-	0.12 (0.05, 1.32)	0.12 (0.05, 1.30)
	Gambela		-	0.03 (0.01, 0.07)^a^	0.03 (0.01, 0.08)^b^
	Harari		-	0.06 (0.02, 0.19)^a^	0.06 (0.02, 0.18)^b^
	Addis Ababa		-	0.06 (0.02, 0.19)^a^	0.58 (0.02, 0.18)
	Dire Dawa		-	0.02 (0.01, 0.06)^a^	0.02 (0.01, 0.06)^b^
	Tigray			1	1
Residency	Rural	-	-	1.11 (0.63, 1.95)	1.42 (0.80, 2.51)
	Urban			1	1
AIC		-	26077.19	26178.36	25927.4
LLR		-	−13027.594	−26178.36	−12941.701
ICC		0.687	0.681	0.503	0.502
Variance		0.77	0.75	0.37	0.36

^a^Significant at model B and C. ^b^Significant at model D.

Household heads older than 35 years were 2.5 (AOR: 2.47, 95% CI: 1.04, 3.02) times more likely to be enrolled in CBHIS than household heads younger than 35 years. Households that had media exposure were 1.4 (AOR: 1.35, 95% CI: 1.02, 2.27) times more likely to be enrolled in CBHIS than their counterparts. Household heads with secondary education were 1.3 (AOR: 1.25, 95% CI: 1.89, 2.44) times more likely to enroll in CBHIS than household heads with no education. Households under the poor wealth index were 69% (AOR: 0.31, 95% CI: 0.21, 0.81) less likely to enroll in CBHIS than rich households. Households in Afar, Gambela, Harari, and Dire Dawa regions were 99% (AOR: 0.01, 95% CI: 0.003, 0.03), 97% (AOR: 0.03, 95% CI: 0.01, 0.08), 94% (AOR: 0.06, 95% CI: 0.02, 0.18), and 98% (AOR: 0.02, 95% CI: 0.01, 0.06), respectively, less likely to enroll in CBHIS (Table 3).

### Ordinary least squares and GWR model's comparison

As shown in Table 4, the GWR model was the best-fit model as compared with the OLS model, with a low AIC (3053.37) as compared with the AIC value of 3162.63 in the OLS model. Additionally, the GWR model was best explained by the predictor variables with an adjusted R^2^ value of 66.72% as compared with the adjusted R^2^ value of 37.44% in the OLS model. The variables that had multicollinearity (redundancy) in the GWR model were removed from the GWR model (Supplementary material).

**Table 4:** Model comparison between OLS and GWR model with AIC and adjusted R^2^.

**Values**	**OLS model**	**GWR model**
AIC	3162.63	3053.37
Adjusted R^2^	37.44%	66.72%

### Geographical heterogeneity factors associated with households' enrollment level in the community-based health insurance scheme

In the geographically weighted regression model, explanatory variables such as secondary education status, poor wealth status, and household media exposure were statistically associated with households' enrollment in CBHIS. The significant explanatory variables have a positive and negative effect on the households' enrollment level in CBHIS across the region of Ethiopia. In addition, the strength of the association between heterogeneity predictors and outcome of interest varies geographically.

**Figure 7:**
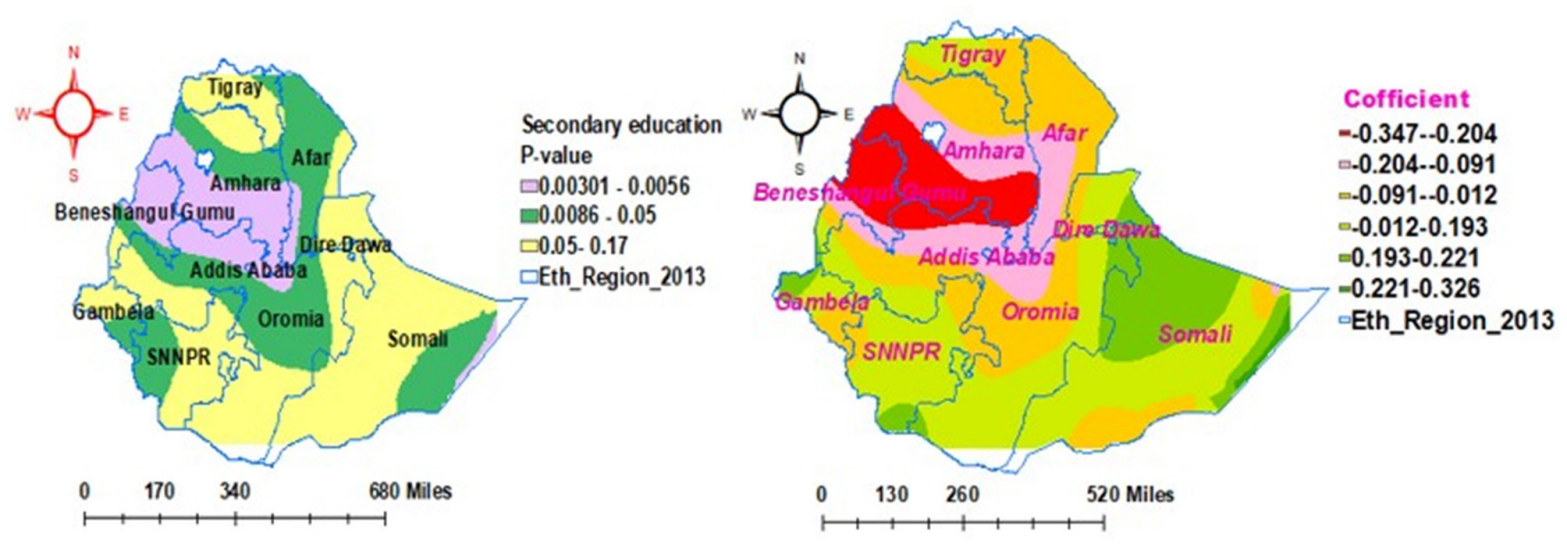
Geographically weighted regression model of households' enrollment level in CBHIS with secondary education status.

As shown in Figure 7, the secondary education status of household heads exhibited different levels of statistical significance in different parts of the country for the enrollment of households in CBHIS. The coefficients of secondary education vary geographically between −0.347 and 0.193 significantly. Keeping all the other predictors constant, the enrollment level of households in CBHIS significantly increased from 34.7% (−0.347) to 80.4% (0.196), with an increase in the number of household heads with secondary education.

**Figure 8:**
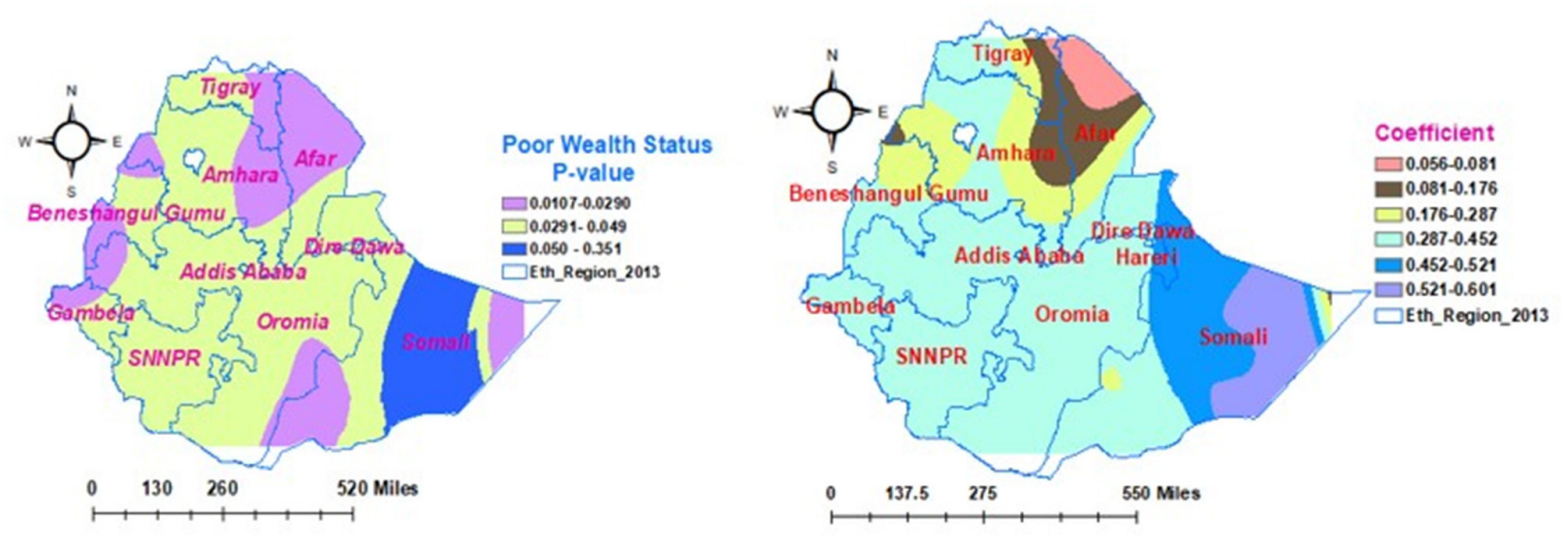
Geographically weighted regression model of households' enrollment level in CBHIS with poor wealth status.

As shown in Figure 8, the poor wealth status of the households exhibited different levels statistical significance in different parts of the country for the enrollment of households in CBHIS. The coefficients of poor wealth status in households vary geographically between 0.056 and 0.452. Keeping all the predictors constant, the households' enrollment level in CBHIS significantly decreased from 45.2% (0.452) to 5.6% (0.056) as their wealth status decreased by one unit.

**Figure 9:**
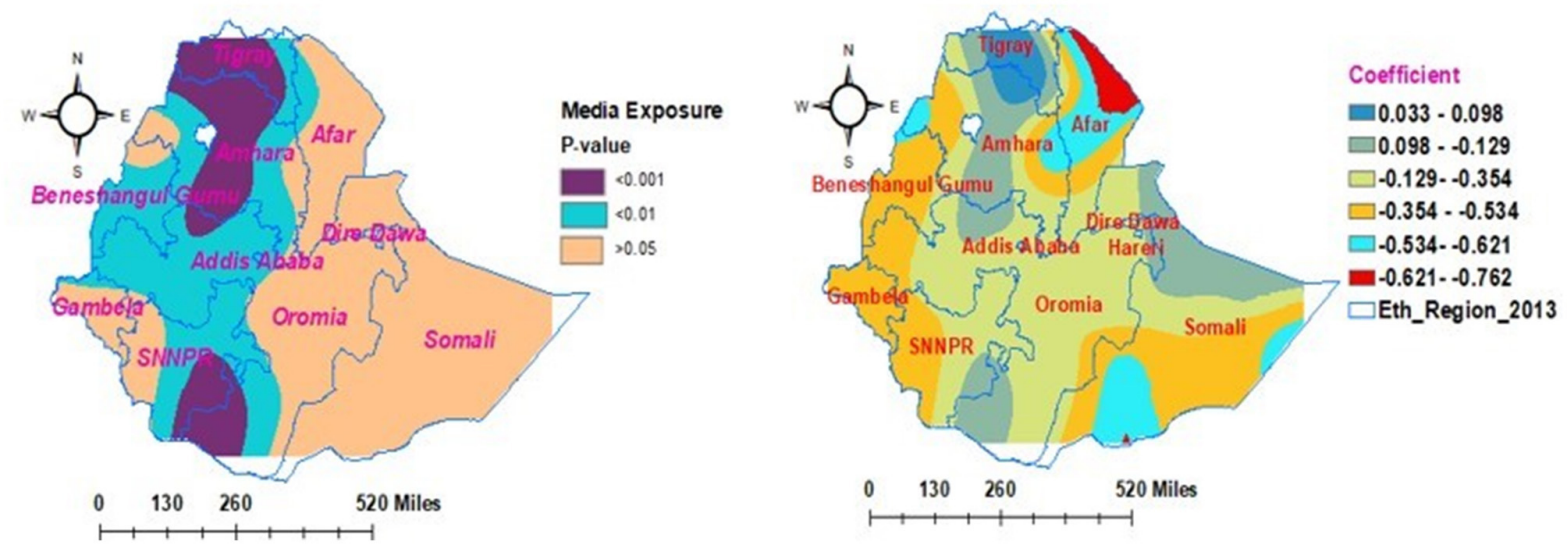
Geographically weighted regression model of households' enrollment level in CBHIS with households' media exposure.

As shown in Figure 9, the households' media exposure had different statistical significance effects in different parts of the country for the households' enrollment in CBHIS. The coefficients of households' media exposure vary geographically and significantly between 0.033 and −0.354. This finding indicated that the media exposure had significant negative and positive effects on households' enrollment level in CBIHS. Keeping all the predictors as constant, as households become more exposed to media, the risk of enrollment level of households in CBHIS decreased from 3.3% (0.033) to 35.4% (−0.354). Except for the vertical intercept of Tigray, Amhara, Oromia, and SNNPR regions, media exposure was not a geographically significant factor in other parts of the country.

## Discussion

In this study, 28.6% of households had a good level of enrollment in CBHIS in Ethiopia. The finding was in line with studies conducted in Nigeria (15.5%) (53), Kenya, where 93% of women have no access to any type of health insurance (54), and Ethiopia (12.8%) (37). However, the evidence indicated lower level of enrollment than the studies conducted in Ethiopia (45%) (55) and Uganda (44%) (56). This difference might be due to household members' compliance with the CBHIS requirements (39), household members' unwillingness to renew their membership (57), and households' inadequate perception of CBHIS (58). In addition, poor government financial support, the high dropout rate from CBHIS (59), lack of clear legislative and regulatory frameworks (54), and an unrealistic enrollment requirement (60) might be possible reasons for the low household enrollment level in CBHIS.

The spatial distribution of households' enrollment level in CBHIS was not random, and the jeopardy of households' enrollment in CBHIS was less likely to occur in Amhara, Tigray, SNNPR, and Oromia regions, whereas high jeopardy of households' enrollment level in CBHIS was more likely observed in Benishangul Gumuz, Gambela, Somalia, Afar, Harari, and Dire Dawa regions. Households in the primary, secondary, and tertiary clusters were more likely to be enrolled in CBHIS than households outside of these clusters. This evidence was strongly supported by a similar spatial study done in Ethiopia (10). This disparity might be due to the difference in access to tertiary-level care and premium payment methods across regions (61).

In the multilevel fixed-effect logistic regression analysis, secondary education status, age of the household head ≥ 35 years, poor wealth status, media exposure, and regions were significantly associated with households' enrollment level in CBHIS. Additionally, geographical heterogeneity factors were assessed according to the GWR analysis report. Thus, predictors such as secondary education status, poor wealth status, and media exposure had significant positive and negative effects on households' enrollment in CBHIS across various geographical areas. The strength of the association between the predictors and outcome of interest was locally independent and varied geographically across the regions of Ethiopia.

The secondary education status contributed to 30% of households' enrollment level in CBHIS. Additionally, the secondary education level status of the household head had the impact of decreasing the jeopardy of households' enrollment level in CBHIS by 45.7%. Additionally, the enrollment level of households in CBHIS significantly increased from 34.7% to 80.4% as the household head's secondary education status increased. This finding indicated that the secondary education of household heads had both negative and positive impacts on the enrollment level of households in CBHIS across the regions of Ethiopia. Moreover, secondary educational status of the household heads was not a geographically significant factor in most parts of Somali, Gambela, SNNPR, Tigray, and southwest parts of Oromia regions. The evidence was in line with studies conducted in Ethiopia (37, 62), Senegal (63), and Asia (64). This evidence might be due to the fact that educated people understand the principles and benefits of CBHIS easily (65) and have more knowledge about the advantages of health insurance. In addition, educated households might be more concerned about their health and insure themselves against unexpected out-of-pocket payments (10).

Having poor wealth status reduces the households' level of enrollment in CBHIS by 69%. Furthermore, the households' enrollment level in CBHIS significantly decreased from 45.2% to 5.6% as their wealth status decreased by one unit. This finding indicates that the poor wealth status of households had different levels of statistical significance in different parts of the country for the enrollment of households in CBHIS. In most parts of the Somali region, poor wealth status was not geographically significantly associated with household enrollment in CBHIS. This finding was in line with studies conducted in Ethiopia (10, 62), Burkina Faso (66), and Ghana (67). This similarity might be because the payment of premium may not be affordable for poor households (68), and high premiums might deter poor households from renewing their membership (69). Moreover, financial constraints and lack of money might be major constraints for households' enrollment in CBHIS (14) in addition to the absence of subsidies in place to cover the premiums (70).

Households exposed to media were 1.4 times more likely to be enrolled in CBHIS. Furthermore, as households become more exposed to media, the risk of enrollment level of households in CBHIS decreased from 3.3% (0.033) to 35.4% (−0.354). This indicated that media exposure had significant negative and positive effects on the enrollment level of households in CBIHS across various geographical areas. The household's media exposure had a significant geographical impact on households' enrollment levels in CBHIS and varied geographically. As households had more media exposure, the households' enrollment level in CBHIS increased by 9.6%. This finding was supported by studies done in Ethiopia (10, 71), Nigeria (72), Uganda (56), and Kenya (54). Since household media exposure has a direct implication for households' access to information about CBHIS, those households that access information about CBHIS directly correlate with good awareness of CBHIS enrollment (57). This correlation might be due to the media creating awareness among communities regarding the principles and implementation of CBHIS (73). Households with good awareness of CBHIS might make informed choices and engage themselves in different knowledge enhancement activities through reading materials (10); individuals with better information may ask for details of the services and get a better understanding of the advantages of CBHIS that drive them to be enrolled (62). Moreover, the health insurance advertisements by health extension workers during their health facility visits also contribute to information about CBHIS (73).

Household heads aged older than 35 years were 2.5 times more likely to be enrolled in CBHIS than household heads who are between 15 and 24 years of age. This evidence was supported by various studies (39, 74, 75). This difference in enrollment might be because older people might be less compliant with CBHIS requirements (39), and they might be less likely to pay membership premiums for shared health insurance than younger people (53).

Households in Afar, Gambela, Harari, and Dire Dawa regions were, respectively, 99%, 97%, 94%, and 98% less likely to be enrolled in CBHIS. This finding was supported by a study that stated that geographical location is a factor in inequality in access to CBHIS (76). Household members in these regions might be less willing to enroll in the CBHIS (77), and health insurance workers and the government in those regions might be less perceived as consistent with insurance activity. Additionally, household members in these regions might have access to other subsidy packages (61).

## Conclusion

Households' enrollment level in the CBHIS is inadequate. Households in Benishangul Gumuz, Gambela, Somalia, Afar, Harari, and Dire Dawa had a high jeopardy of enrollment in CBHIS. Educational status, age of the household heads, wealth status, media exposure, and regions were statistically significant factors for households' enrollment in CBHIS. The secondary education status of household heads, poor wealth status, and media exposure had positive and negative geographical effects on the households' enrollment in CBHIS.

## Recommendation and future research direction

Priority attention needs to be paid to the enrollment of households in cold areas in CBHIS. Enhancing the educational status of household members, providing financial support, and premium subsidies would enhance households' enrollment levels in CBHIS. It is recommended that health policymakers and implementers propose good CBHIS implementation frameworks and packages. Quantitative and qualitative research is needed considering contextual, technological, behavioral, and structural factors of households' enrollment level in CBHIS.

## Limitations and strengths of the study

A cross-sectional study may have a temporal relationship between the outcome and independent variables. The data for EMDHS were gathered based on the respondents' recall ability, which might be subjected to recall bias. Moreover, important variables that can significantly determine the households' enrollment level in CBHIS may not be included as limited variables are found in the 2019 EMDHS data sets. Despite these limitations, the data for the study were collected across the country, and the results can be nationally representative. Furthermore, multilevel analysis was employed, which is more appropriate for cluster data to solve data dependency. The geographically weighted regression analysis was performed. The findings and various data analysis techniques used in this study could be used as inputs in future research.

## Data availability statement

The original contributions presented in the study are included in the article/Supplementary material, further inquiries can be directed to the corresponding author.

## Ethics statement

Ethical approval was not required for the studies involving humans because this study was based on secondary data source, which is publicly accessible from The Measure Demographic and Health Survey website. Thus, direct ethical approval from the ethical review board was not necessary. The studies were conducted in accordance with the local legislation and institutional requirements. Written informed consent for participation was not required from the participants or the participants' legal guardians/next of kin in accordance with the national legislation and institutional requirements because this study was based on secondary data source, which is publicly accessible from The Measure Demographic and Health Survey website. Thus, direct consent from the study participants was not necessary.

## Author contributions

AWD: Conceptualization, Data curation, Formal analysis, Investigation, Methodology, Validation, Visualization, Writing – original draft, Writing – review & editing.
