# Supplementary material for: Spatial distribution and geographical heterogeneity factors associated with households' enrollment level in community-based health insurance
Source: Front Public Health. 2024 May 17;12:1305458. doi: 10.3389/fpubh.2024.1305458 (PMC11140031; doi:10.3389/fpubh.2024.1305458)
Supplement: Supplementary file 1 [file Table_1.DOCX]

Multicollinearity check to identify redundant explanatory variables

| **Explanatory variables** | **VIF** | **remark** |
| --- | --- | --- |
| Poor wealth status | 0.567 | Accepted for GWR |
| Middle wealth status | 0.871 | Removed for GWR |
| Rich wealth status | 0.687 | Accepted for GWR |
| No educational status | 0.797 | Removed for GWR |
| Primary educational status | 0.634 | Accepted for GWR |
| Secondary educational status | 0.498 | Accepted for GWR |
| Higher educational status | 0.312 | Accepted for GWR |
| 15-24 years of age | 0.804 | Removed in GWR |
| 25-34 years of age | 0.784 | Removed for GWR |
| >=35 years of age | 0.668 | Accepted for GWR |
| Households exposed to media | 0.576 | Accepted for GWR |
| Households not exposed to media | 0.789 | Removed for GWR |
| Male household head | 0.421 | Accepted for GWR |
| Female household head | 0.404 | Accepted for GWR |
| Urban resident | 0.907 | Removed for GWR |
| Rural resident | 0.915 | Removed for GWR |
